# Interaction between bacterial adhesins leads to coaggregation by the oral bacteria *Veillonella parvula* and *Streptococcus gordonii*

**DOI:** 10.1128/mbio.03279-24

**Published:** 2025-01-10

**Authors:** Jack C. Leo

**Affiliations:** 1 Antimicrobial Resistance, Omics and Microbiota Group, Centre for Systems Health and Integrated Metabolic Research, Department of Biosciences, Nottingham Trent University, Nottingham, United Kingdom; Gulbenkian Institute for Molecular Medicine, Oeiras, Portugal

**Keywords:** *Veillonella parvula*, coaggregation, *Streptococcus gordonii*, oral biofilm, trimeric autotransporter adhesin

## Abstract

*Veillonella parvula* is an unusual diderm firmicute that plays a central role in the formation of dental biofilm formation through coaggregation with many other oral bacteria. However, the molecular interactions leading to oral biofilm formation are largely unknown. In a recent study (L. Dorison, N. Béchon, C. Martin-Gallausiaux, S. Chamorro-Rodriguez, et al., mBio 15:e02171-24, 2024, https://doi.org/10.1128/mbio.02171-24), coaggregation by *V. parvula* was shown to be mediated by trimeric autotransporter adhesins (TAAs), which are large, fibrous surface proteins widespread in Gram-negative bacteria. Importantly, this study identified the binding partner protein on a coaggregating bacterium, *Streptococcus gordonii*, which the authors called VisA. This finding is the first time a TAA mediating coaggregation with a different type of protein has been established and suggests that specifically interacting protein partners may have coevolved multiple times to allow complex biofilm formation, as exemplified by the development of dental plaque. Understanding these interactions might lead to innovations to reduce build-up of dental plaque and associated oral diseases.

## COMMENTARY

*Veillonella* spp. are common, obligately anaerobic members of the oral microbiota belonging to the phylum Bacillota (formerly Firmicutes). Most members of this phylum have a classically Gram-positive cell structure; however, veillonellae (and other members of the class Negativicutes) are atypical in this group, as they have a diderm cell envelope, i.e., having an outer membrane similar to classical Gram-negative bacteria, though with some notable differences in biogenesis and homeostatic mechanisms (1, 2). Veillonellae contribute to the formation of dental plaque by binding to early colonizers of the tooth surface (mostly streptococci) and then recruiting late colonizers to the forming biofilm, including pathogenic species like *Porphyromonas gingivalis* and *Treponema denticola*. Veillonellae can, therefore, be considered ecological keystone species important for oral biofilm formation (3). The ability of veillonellae to bridge the early and late plaque colonizers depends on the phenomenon of coaggregation, where microbes of different species bind to each other through mutually attractive interactions (4). In the case of veillonellae, coaggregation is beneficial, as these organisms then metabolize the lactate produced by their coaggregating partners.

How veillonellae coaggregate with other bacteria has been unclear. Until recently, dissecting the coaggregation mechanisms of veillonellae has been hampered by the lack of genetically tractable strains. However, some strains of *Veillonella parvula*, including the strain SKV38, are naturally competent, and this has allowed manipulation of the SKV38 genome. In a recent study (5), Dorison et al. from the Institut Pasteur investigated the coaggregation properties of *V. parvula* SKV38. Previously, the group had identified nine genes coding for trimeric autotransporter adhesins (TAAs) in its genome (6). TAAs are large, fibrous, homotrimeric cell-surface proteins with modular domain compositions that mediate attachment to a variety of surfaces and molecules (7). In addition, TAAs often mediate autoaggregation (clumping of the same species of microbe) and biofilm formation (8, 9). The prototype of this protein family is the *Yersinia* adhesin YadA, which has a variety of functions, including binding to collagen and other extracellular matrix molecules, autoaggregation, and biofilm formation, as well as mediating serum resistance (10).

In *V. parvula* SKV38, autoaggregation is mediated by the TAA VtaA (6). The genes for the eight other TAAs encoded by this strain (VtaB through VtaI) are all localized in a single genomic adhesin cluster. Through deleting individual or multiple TAA genes, Dorison et al. show that VtaA also mediates coaggregation with *Streptococcus oralis*, whereas VtaA and VtaE are involved in coaggregation with *Actinomyces oris*, and VtaE and VtaD are needed for coaggregation with *Streptococcus gordonii*. However, the TAAs of *V. parvula* do not appear to mediate adhesion to host cells, in contrast to the TAA Hag1 of *V. atypica* OK5 (11), which is encoded by the positional orthologue of *vtaA* but is structurally distinct from VtaA.

**Fig 1:**
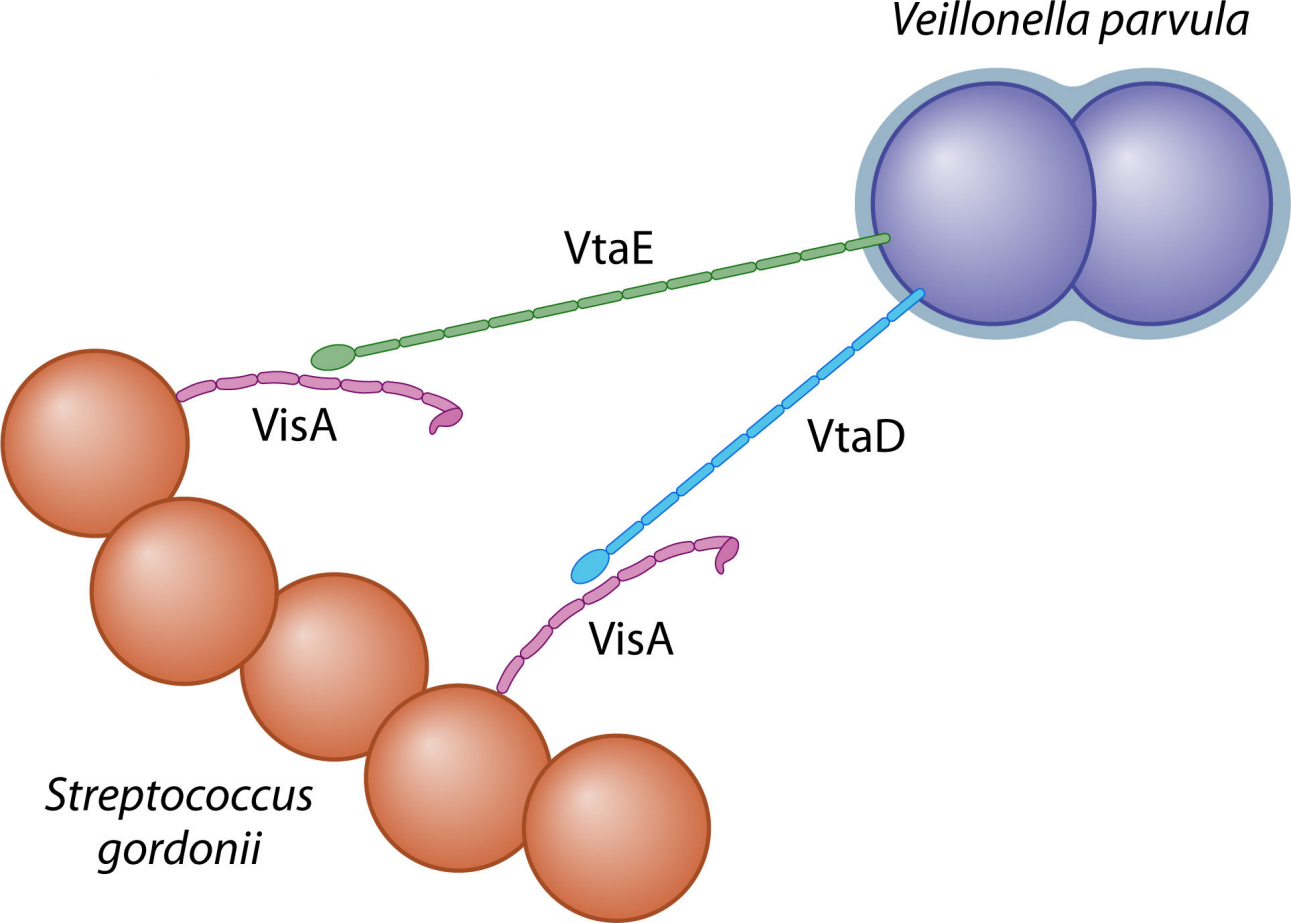
Two TAAs of *Veillonella parvula* SKV38 mediate binding to a surface protein of *S. gordonii*. Schematic representation of how VtaE (green) and VtaD (blue) mediate coaggregation by binding to VisA (magenta) of *S. gordonii*. The binding sites in the figure for VtaE and VtaD are purely illustrative; the exact binding sites are currently unknown. The unstructured C-terminus of VisA has been omitted in the figure. This figure was inspired by Fig. 7 in reference 5.

The main finding of the study by Dorison et al. was to identify the partner protein of *S. gordonii* to which both VtaE and VtaD bind. Coaggregation between different enterobacterial TAAs has been observed *in vitro* (12), but this is the first time coaggregation between a TAA and a different type of bacterial protein has been demonstrated. Previous work showed that Hag1 of *V. atypica* OK5 mediated coaggregation with a variety of oral bacteria, but the exact binding partner proteins were not identified (11), although the *S. gordonii* protein Hsa was suspected as a partner for Hag1 (13). Dorison et al. were able to identify a sortase-assembled adhesin, which they called VisA, as the binding partner for VtaE and VtaD. Like the TAAs, VisA is an extended, fibrous protein that might also mediate adhesion to surfaces (Fig. 1). VtaE appears to be the primary coagglutinin for binding to VisA, as VtaD only mediated low levels of coaggregation. VtaE and VtaD are similar in sequence, but VtaE is ~100 nm longer than VtaD based on Alphafold predictions (Fig. 1). The masking of VtaD by VtaE or other surface structures could explain why the shorter adhesin does not play as large a role in coaggregation to *S. gordonii*.

The coaggregation of *S. gordonii* and *V. parvula* leads to biofilm formation. Normally, the coaggregating bacteria form fully mixed biofilms. However, when coaggregation was prevented by deleting either *visA* in *S. gordonii* or *vtaDE* in *V. parvula*, the resulting biofilms were segregated, demonstrating that coaggregation is required for normal biofilm development.

Dorison et al. also investigated the effect of coaggregation on the transcriptomic response of *V. parvula* and *S. gordonii*. Remarkably, coaggregation had very little impact on the transcriptomic profiles of the bacteria in comparison to coculture in the absence of coaggregation. Thus, coaggregation as such does not lead to major changes in gene expression for *V. parvula*, suggesting that coaggregation may be more a way of ensuring proximity to nutrient sources for efficient growth rather than a major shift in lifestyle.

The study by Dorison et al. is a first step in dissecting the complex interactions of the oral microbiota at the molecular level. The next steps would be to determine how VisA and VtaD/E interact: which domains of the proteins are important for binding and what do these complexes look like? Although this study shows a few *V. parvula* TAAs are involved in coaggregation, the role of the others is still open. They may mediate coaggregation with yet other, untested species, be involved in the colonization of non-oral environments, for example, the gut, or possibly even play a protective role against antimicrobial compounds in the saliva or oral surfaces. Another question concerns the function of VisA. Presumably, this protein has some other activities than just mediating coaggregation. It will be interesting to discover other binding partners for *V. parvula* TAAs, as well as elucidate the primary functions of these adhesion proteins. Investigating these functions and interactions will undoubtedly prove to be a fruitful area of research to decipher the complexities of oral biofilm development, and ultimately this research could lead to innovations promoting oral health.
